# Serum Amyloid A in the Placenta and Its Role in Trophoblast Invasion

**DOI:** 10.1371/journal.pone.0090881

**Published:** 2014-03-10

**Authors:** Silvana Sandri, Alexandre Urban Borbely, Isabella Fernandes, Edson Mendes de Oliveira, Franciele Hinterholz Knebel, Rodrigo Ruano, Marcelo Zugaib, Fabiola Filippin-Monteiro, Estela Bevilacqua, Ana Campa

**Affiliations:** 1 Departamento de Análises Clínicas e Toxicológicas, Faculdade de Ciências Farmacêuticas, Universidade de São Paulo, São Paulo, Brazil; 2 Departamento de Biologia Celular e do Desenvolvimento, Instituto de Ciências Biomédicas, Universidade de São Paulo, São Paulo, Brazil; 3 Departamento de Cirurgia, Faculdade de Veterinária e Zootecnia, Universidade de São Paulo, São Paulo, Brazil; 4 Departamento de Obstetrícia e Ginecologia, Faculdade de Medicina, Universidade de São Paulo, São Paulo, Brazil; 5 Departamento de Análises Clínicas, Universidade Federal de Santa Catarina, Florianópolis, Brazil; VU University Medical Center, Netherlands

## Abstract

The serum amyloid A (SAA) protein is known to function in the acute phase response and immunoregulation. Recently, SAA has been shown to be involved in cell proliferation, differentiation and migratory behavior in different cell types. Here, we evaluated whether exogenous SAA could influence trophoblast invasion and differentiation using both the trophoblast-like BeWo cell line and fully differentiated human extravillous trophoblast cells (EVT) isolated from term placentae. SAA stimulated BeWo cell invasion, as measured in Matrigel invasion assays, and induced metalloprotease mRNA expression and activity. Given that BeWo cells express Toll-like receptor 4 (TLR4), a known receptor for SAA, we examined the role of TLR4 in SAA-induced invasion using a TLR4 neutralizing antibody. We also tested whether SAA could affect markers of trophoblast syncytialization in BeWo cells. We observed that SAA decreased βhCG secretion and did not influence trophoblast syncytialization. Using EVT cells isolated from human term basal plates, we confirmed that SAA at 1 and 10 µg/mL doubled EVT invasion in a TLR4-dependent manner, but at 20 µg/mL inhibited EVT cells invasiveness. In addition, we observed that SAA was expressed in both BeWo cells and human term placentae, specifically in the syncytiotrophoblast, decidual cells and EVT. In conclusion, SAA was identified as a molecule that functions in the placental microenvironment to regulate metalloprotease activity and trophoblast invasion, which are key processes in placentation and placental homeostasis.

## Introduction

Serum amyloid A (SAA) is encoded by the four human SAA gene isoforms (*SAA1–4*). *SAA1* and *SAA2* encode acute phase proteins (A-SAA), while *SAA4* is constitutively expressed (C-SAA), and *SAA3* is a pseudogene [1]. SAA is primarily synthesized by hepatocytes [1], and its extra-hepatic sources include leukocytes [2], adipocytes [3], synoviocytes [4], tumor cells [5] and first trimester trophoblast cells [6]. SAA has been shown to play biological roles in lipid metabolism [7], immunomodulation [8]–[10] and cell proliferation [11], [12] and invasion [13].

Trophoblast cells, as a key constituent of the human placenta, play a fundamental role in successful pregnancy. These cells are fated to become either villous cytotrophoblast cells, which proliferate and then differentiate via fusion to form the syncytiotrophoblast, or invasive extravillous cytotrophoblast cells (EVT), which form from proliferating cells streaming out of the syncytiotrophoblast and ultimately differentiate into a multilayered cell column [14]. These cells then proceed to detach from the column and invade the newly formed decidua, where the maternal vascular system is remodeled, establishing the maternal–fetal circulation. It is widely accepted that the invasion of EVT cells into the decidua is controlled by a series of tightly regulated intercellular signaling events mediated by growth factors, cytokines, hormones and other molecules [15]. EVT invasion is facilitated by the degradation of the endometrium/decidua extracellular matrix by various proteases, such as metalloproteases (MMPs) [16]. Insufficient migration and shallow invasion into the maternal decidua are linked to recurrent spontaneous abortion, fetal intrauterine growth restriction and pre-eclampsia [17]. However, our understanding of the mechanisms and molecules involved in this process remains incomplete.

The expression of SAA in first trimester trophoblast has been speculated to be related to SAA-induced immunoregulatory effects [18], and no other function of this protein in the placental microenvironment has previously been identified. In this study, the effects of SAA on cell invasion and differentiation in a trophoblastic lineage were evaluated using BeWo cells. Furthermore, to identify potential roles of SAA in a fully functional placenta, we took advantage of a working experimental model of EVT cells isolated from human term basal plates [19]. We determined that SAA induced BeWo and EVT cell invasion through a process that was dependent on the Toll-like receptor 4 (TLR4).

## Materials and Methods

### Reagents

Bovine serum albumin (BSA), collagenase type II and forskolin were supplied by Sigma Chemical Co. (St. Louis, MO, USA). Amphotericin B, deoxyribonuclease (DNase) type I, Dulbecco’s Modified Eagle’s Medium: Nutrient Mixture F-12 (DMEM/F12), fetal bovine serum (FBS), gentamicin, Icoveco Modified DMEM Medium (IMDM), penicillin, streptomycin and the Trizol reagent were purchased from Invitrogen (Carlsbad, CA, USA). Matrigel and transwell inserts were obtained from Becton Dickinson (Franklin Lakes, NJ, USA). rSAA was purchased from Peprotech Inc. (Rocky Hill, NJ, USA). According to the supplier, the amount of endotoxin contaminant is lower than 0.1 ng/1 µg protein, and purity is greater than 98%, as assessed by SDS-PAGE gel and HPLC analyses. All other reagents used came from Merck (Darmstadt, Germany) unless otherwise indicated.

### Cell Culture

The human BeWo choriocarcinoma cell line was obtained from Banco de Células do Rio de Janeiro (Brazil). The cells were maintained in DMEM/F12 supplemented with 10% FBS, 100 IU/mL penicillin and 100 µg/mL streptomycin at 37°C in a humidified atmosphere with 5% CO_2_.

### Matrigel Invasion Assay

Transwell membranes coated with Matrigel (BD Biosciences, San Jose, CA) were used for *in vitro* invasion assays. BeWo cells (1.0×10^5^ cells/well) or freshly isolated EVT cells (5.6×10^4^ cells/well) were plated in the upper chamber in DMEM/F12 supplemented with 2% FBS. SAA was added to the medium in the upper chamber at different concentrations. Following a 48 h incubation period, the medium was aspirated from the top and bottom wells, and non-invading cells were removed from the top well with a cotton swab. The remaining cells were fixed with 3% paraformaldehyde and stained with 0.2% crystal violet prior to counting under an inverted light microscope (Axiovert S100, Zeiss, Germany). In each well, seven independent 20× fields were counted for quantitation. To evaluate the involvement of TLR4 in SAA-mediated cell invasion, BeWo and EVT cells were plated in the upper chamber in DMEM/F12 supplemented with 2% FBS and pre-incubated with 10 µg/mL of an anti-human TLR4 antibody (R&D systems, Minneapolis, USA) for 1 h. Following incubation, the cells were stimulated with SAA (10 µg/mL) and maintained in culture for 48 h. The concentration of the TLR4 antibody was defined based on profiling the inhibition of TNF-α release induced by lipopolysaccharide (LPS) from *Escherichia coli* 0111:B4 (Sigma-Aldrich, St. Louis, MO, USA). The TLR4 antibody was able to inhibit the LPS-induced TNF-α release from mononuclear cells by 70% at a concentration of 10 µg/mL (data not shown).

### RNA Extraction and Reverse Transcription

Total RNA was extracted from BeWo cells and placental tissue using the RNeasy Mini kit (Qiagen, Hilden, Germany) or Trizol reagent (Invitrogen, Carlsbad, CA, USA), respectively, in accordance with the instructions of the supplier. A total of 1 µg of RNA, previously treated with DNase I (Invitrogen, Carlsbad, CA, USA), was retrotranscribed into cDNA using the High Capacity RNA to cDNA kit (Life Technologies, Carlsbad, CA, USA) according to the manufacturer’s instructions.

### Quantitative Real-time PCR

The following specific primers were used: SAA1 (forward 5′-CCTGGGCTGCAGAAGTGATCAGCGA-3′ and reverse 5′-AGTCCTCCGCACC-ATGGCCAAAGAA-3′) (NM_199161.2); SAA2 (forward 5′-CTGGGCCGCAGAAGTGA-TCAGCA-3′ and reverse 5′-GAGTCCTCCGCACCATGGCCTGT-3′) (NM_030754.3); SAA4 (forward 5′-GTTCGTTTTTCAAGGAGGCTCTCCAA-3′ and reverse 5′-GGATATCATTATGTCCCAATAGGCTCT-3′) (NM_006512.2); MMP-2 (forward 5′-GACTACGACCGCGACAAGA-3′ and reverse 5′-TGTTGCCCAGGAAAGTGAA-3′) (NM_004530.4); MMP-9 (forward 5′-GAGGTGGACCGGATGTTCC-3′ and reverse 5′-AACTCACGCGCCAGTAGAAG-3′) (NM_004994.2); Toll-like 4 (forward 5′-AAGCCGAAAGGTGATTGTTG-3′; reverse 5′-CTGAGCAGGGTCTTCTCCAC-3′) (NM_003266); and tubulin (forward 5′-TCAACACCTTCTTCAGTGAAACG-3′ and reverse 5′-AGTGCCAGTGCGAACTTCATC-3′) (NM_006082.2), which was analyzed as a constitutively expressed control. BLAST searches were conducted on all primer sequences to ensure gene specificity. Quantitative RT-PCR was performed using the Gene AMP 7500 Sequence Detection System (PE Applied Biosystems, Foster City, CA, USA), with SYBR Green Master mix (Applied Biosystems, Mount Holly, NJ, USA). Each PCR mixture contained 1 ng of cDNA, 6 µL of SYBR Green master mix and 3 µL of forward and reverse primers at concentration of 600 nM. The reaction conditions were as follows: 95°C for 10 min, followed by 40 cycles at 95°C for 10 s (melting) and 60°C for 1 min (annealing and elongation). The cycle threshold (Ct) for each run was set to 0.1 when amplification was observed in log phase. Relative gene expression was determined using the ΔΔCt method. The efficiency of each reaction was validated as previously described [20].

### Measurement of MMP-2 and MMP-9 Activities

BeWo cells were cultured at a density of 2.5×10^5^ cells/well in 24-well plate in DMEM/F12 supplemented with 10% of FBS for 18 h. After this incubation period, the supernatant was discarded, and the adherent cells were washed twice with DMEM/F12 containing 1% BSA, then incubated for 24 h with DMEM/F12 containing 1% BSA. Subsequently, the medium was replaced, and the cells were stimulated with SAA (10 µg/mL) for 48 h. At the end of this incubation period, the supernatant was harvested and centrifuged at 400×g for 10 min at 4°C, then immediately stored at −80°C for later analysis. The activities of MMP-2 and MMP-9 were determined using the Biotrak Activity Assay System (MMP-2 Biotrak Activity Assay RPN 2631; MMP-9 Biotrak Activity Assay RPN2634, GE Healthcare, Buckinghamshire, UK), following the instructions of the supplier. The adherent cells were also collected to measure protein concentration, which was used for data normalization.

### Flow Cytometry Analysis

BeWo cells were incubated in 5% normal goat serum (Vector Laboratories, Burlingame, CA, USA) for 30 min. After washing in 0.1% PBS/BSA, the cells were fixed and permeabilized with commercial buffers (e-Bioscience, San Diego, CA, USA) according to the instructions of the supplier. The BeWo cells were then incubated with anti-SAA (Abcam, Cambridge, UK), previously conjugated with the Zenon Tricolor mouse IgG labeling kit, in permeabilization buffer. Finally, the cells were washed, and the cellular pellet was resuspended in PBS/BSA 0.1% and analyzed. To detect TLR4 protein expression, BeWo cells were incubated for 30 min with a PE-labeled monoclonal antibody against human TLR4 (R&D systems, Minneapolis, USA). The stained cells were analyzed using FACSCanto flow cytometry equipment (Becton Dickinson, San Diego, CA, USA). The obtained data were analyzed using FlowJo software (Tree Star Inc., Ashland, OR, USA).

### βhCG Secretion

BeWo cells were cultured in IMDM supplemented with 2% FBS and SAA (1, 10 or 20 µg/ml) or forskolin (50 µM) for 72 h. The concentration of βhCG in the culture medium was measured based on automated immunochemiluminescence (ADVIA Centaur CP, Siemens, Erlangen, Germany) at the Laboratório de Análises Clínicas/HU/USP. To compare the secretion of βhCG in the supernatants, the results were normalized to 1 µg of total protein.

### F-actin Labeling

The F-actin was labeled with rodhamine-phaloidin to evaluate the formation of multinucleated cells. 5×10^3^ BeWo cells were plated using the Nunc Lab-Tek II Chamber Slide System (Thermo Scientific, Waltham, MA, USA). Following overnight incubation, the cells were treated with 20 µg/mL SAA in IMDM supplemented with 2% of FBS, then incubated for an additional 72 h; 50 µM forskolin was used as a positive control. The supernatant was subsequently collected, and the cells were fixed with a 3.7% (v/v) formaldehyde solution in PBS (pH 7.4) for 10 min, followed by permeabilization for 5 min with a 0.2% Triton X-100 solution. Finally, the cells were labeled with rhodamine-phalloidin (Invitrogen, Carlsbad, CA, USA) and 0.2 µM DAPI (Invitrogen, Carlsbad, CA, USA).

### Tissue Collection and Isolation of Extravillous Trophoblasts from Term Basal Plates

Full-term placentae (37–40 weeks of gestation, n = 15) were obtained from women undergoing elective Caesarean section who had healthy babies and did not exhibit any complications during pregnancy. The signed patient consent was obtained prior to delivery. Ethical committee approvals for this study were granted by the Hospital Universitário and the Faculdade de Ciências Farmacêuticas from Universidade de São Paulo following the norms as per Declaration of Helsinki. Patients were handed an information sheet telling them about the study with consent sheet, which were also approved by ethics committee. All signed consent sheets were stored in case of the need for audit. The cotyledons were maintained in PBS (pH 7.4) supplemented with 1% streptomycin/penicillin, 0.5% gentamicin, 0.25% amphotericin B, and 25 mM glucose on ice until further processing. The term basal plate is a thin membrane with a thickness of 3–6 mm, which was carefully dissected from the villous tissue and processed according to Borbely et al. [19]. Briefly, the obtained fragments were coarsely minced, and approximately 5 g of wet tissue was incubated for 1 h with 20 mL DMEM/F12 containing 4% bovine serum albumin (BSA), 125 U/mL collagenase type II and 25 U/mL DNase type I at 37°C in a water bath, followed by the addition of 20% FBS. The resulting cell suspension was double-filtered, first through a 100 µm mesh and then through a 70 µm mesh. The suspension was then centrifuged at 400×g, and the cells were washed and resuspended in complete DMEM/F12 (supplemented with 1% antibiotics, 10% FBS, 0.01% insulin, 520 µg/mL calcium lactate, 56 µg/mL sodium pyruvate and 1% nucleosides). EVT cells were isolated using a gradient of 30 and 60% Percoll (GE Healthcare, Uppsala, Sweden), followed by centrifugation at 700×g for 30 min [21]. EVT cells were collected from the top of the 30% gradient. When necessary, the cell suspension was incubated with 0.83% ammonium chloride in PBS and centrifuged at 400×g for 5 min to remove blood cells.

### Immunolocalization of SAA

Tissue sections from the fetal and maternal regions of term placentae (4 µm thickness), fixed in 4% paraformaldehyde, were deparaffinized and rehydrated for immunohistochemical and immunofluorescence analysis. In the immunohistochemistry analysis, a mouse monoclonal antibody against SAA-1 (Abcam, Cambridge, ENG, UK) and a negative control mouse IgG (Dako, Glostrup, Denmark) were used. Bound antibodies were visualized using the Sigma Fast DAB (3, 3′-diaminobenzidine) staining kit (Sigma Chemical Co., St. Louis, MO, USA), according to the manufacturer’s instructions. The antibody used in these assays was able to identify the SAA1 and SAA2 isoforms protein. Tissue sections were counterstained using hematoxylin (Merck, Darmstadt, Germany) and mounted. In the immunofluorescence analysis, the tissue sections were blocked with 1% BSA in PBS, followed by incubation with an anti-SAA antibody and cytokeratin-7 (CK7) (Dako, Glostrup, Denmark) previously conjugated with the Zenon Tricolor mouse IgG labeling kit (Invitrogen, Carlsbad, CA, USA). Nuclei were stained with 0.2 µM DAPI (Invitrogen, Carlsbad, CA, USA), and coverslips were finally mounted using 1∶9 (v/v) PBS/glycerol.

### Photographic Documentation

An Axiovert S100 inverted light microscope (Zeiss, Jena, Germany) was employed for general cell morphology and cell counting. Immunofluorescence was analyzed using an Axioskop 2 fluorescence microscope (Zeiss), and images were captured using the program AxioVision 4.7 (Zeiss).

### Statistical Analysis

Statistical analyses were performed using Graph Pad Prism4 (Graph Pad Software Inc., San Diego, CA, USA). When multiple samples were compared with one independent variable, one-way analysis of variance with the Newman-Keuls *post hoc* test was performed. The level of significance was set at *p*<0.05.

## Results

### SAA Induced Trophoblast Invasion

**Figure 1:**
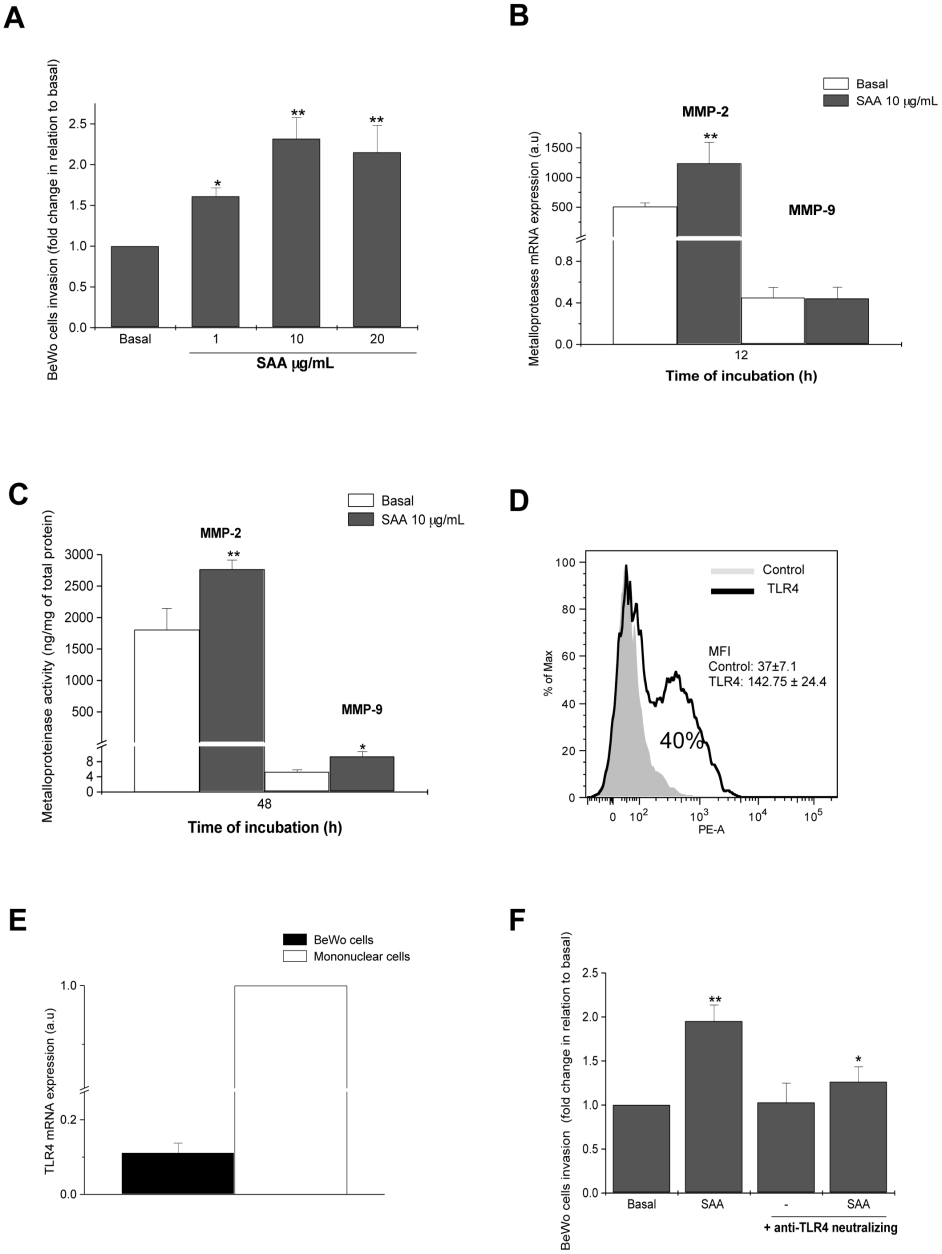
Effects of SAA in BeWo cells. (A) SAA-induced BeWo cell invasion in a Matrigel-coated transwell assay cultured for 48 h. (B, C) SAA induced metalloprotease mRNA expression and activity. *p<0.05 and **p<0.01 vs. basal. (D) TLR4-positve BeWo cells. (E) TLR4 mRNA expression. (F) Inhibitory effect of a TLR4-neutralizing antibody on SAA-induced BeWo cell invasion. *p<0.05 and **p<0.01 vs. treatment with the TLR4-neutralizing antibody and basal, respectively. The data represent the mean ± SE of four independent experiments performed in duplicate.

The effects of SAA on BeWo cell invasion were evaluated based on Matrigel-coated transwell assays, mRNA expression analysis and the activity of metalloproteases. The invasiveness of BeWo cells was increased in the presence of SAA depending on SAA concentration up to 10 µg/mL and maintained up to 20 µg/mL (**Fig. 1A**). Furthermore, SAA treatment increased the expression of MMP-2 mRNA but did not affect the expression MMP-9 mRNA at 12 h of incubation (**Fig. 1B**). SAA increased the activities of both MMP-2 and -9, although the activity of MMP-9 was lower than that of MMP-2 at 48 h of culture (**Fig. 1C**). Given that TLR4 is one of the known receptors for SAA [10], we evaluated the potential role of this receptor in SAA-induced invasion. First, we demonstrated the expression of TLR4 in BeWo cells. Through flow cytometric analysis, it was found that approximately 40% of BeWo cells were positive for TLR4, showing a median fluorescence intensity that was 5-fold higher than the control (**Fig. 1D**). The cells also expressed TLR4 mRNA, at approximately 15% of the TLR4 mRNA expression level observed in human mononuclear cells (**Fig. 1E**). To examine the involvement of TLR4 in SAA-induced invasion, we used a commercially available anti-TLR4 neutralizing antibody. BeWo cells were pre-treated for 1 h, followed by stimulation with SAA. Treatment with the anti-TLR4 neutralizing antibody alone did not affect the invasiveness of BeWo cells in relation to the control, whereas SAA-induced BeWo cell invasion was decreased by approximately 50% by the addition of anti-TLR4 compared to invasion induced by SAA alone (**Fig. 1F**).

**Figure 2:**
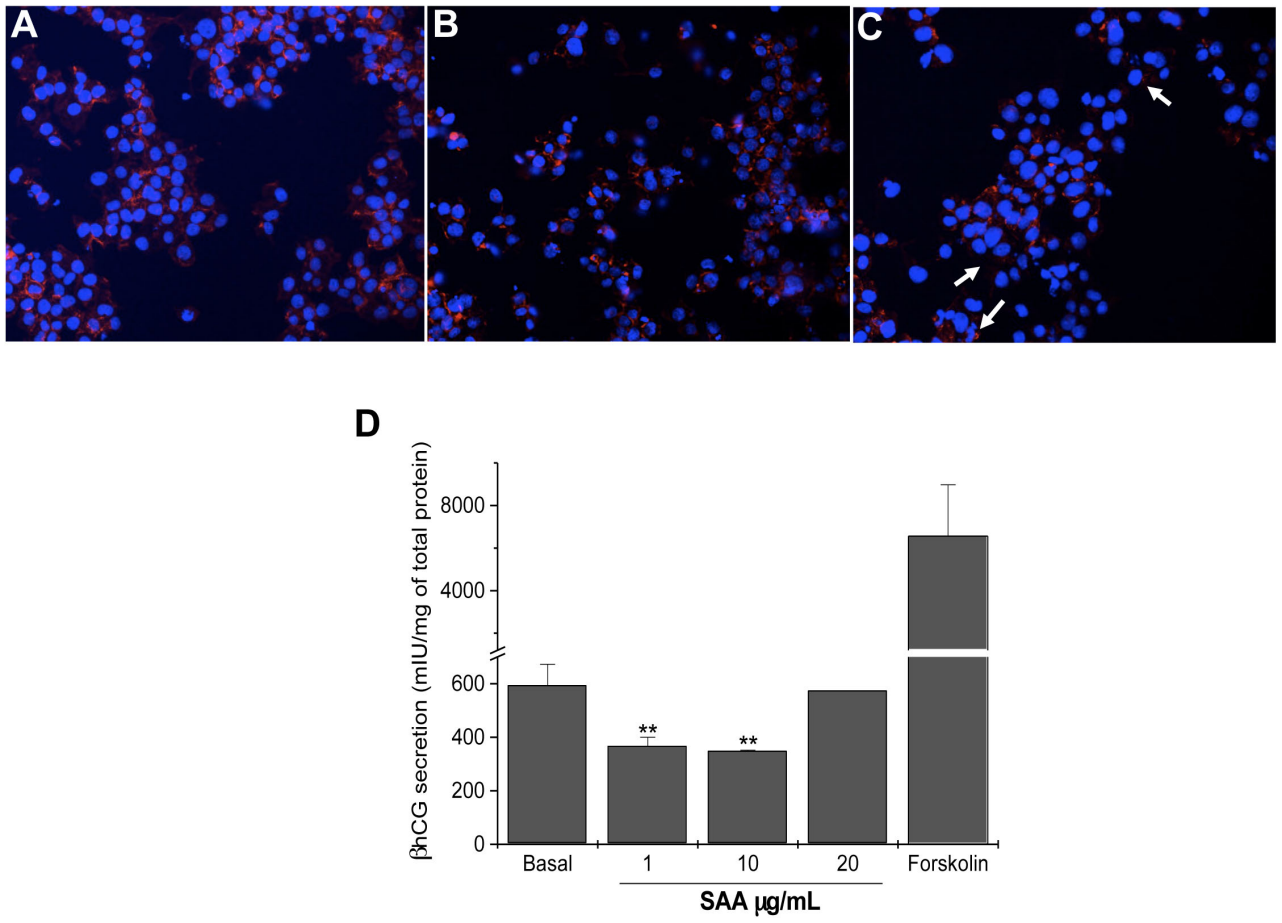
Effects of SAA on syncytialization. (A–C) SAA did not affect the fusion of BeWo cells as observed by F-actin staining (rhodamine-phalloidin). (A) BeWo cells untreated, (B) treated with 20 µg/mL SAA (C) and 50 µM forskolin, a positive control, were incubated for 72 h. Nuclei were stained with DAPI. arrows- multinucleated syncytiotrophoblasts. 200×magnification. (D) βhCG secretion was decreased by the presence of SAA. BeWo cells were treated with SAA at different concentrations and 50 µM forskolin, a positive control, and cultured for 72 h. The data represent the median ± SE of four independent experiments performed in duplicate. **p<0.01 vs. basal.

As BeWo cells are also used to study the syncytialization process, we verified the effects of SAA on morphology and the secretion of βhCG, a biochemical marker of syncytialization [22]. Morphological differentiation was assessed using F-actin staining with rodhamine-phalloidin to distinguish undifferentiated cells from multinucleated syncytiotrophoblasts (three or more nuclei). It was observed that SAA did not significantly increase the number of multinucleated cell compared with either the basal or forskolin (**Fig. 2A–C**). SAA inhibited by 40% the secretion of βhCG at concentrations of 1 and 10 µg/mL but not at 20 µg/mL (**Fig. 2D**).

**Figure 3:**
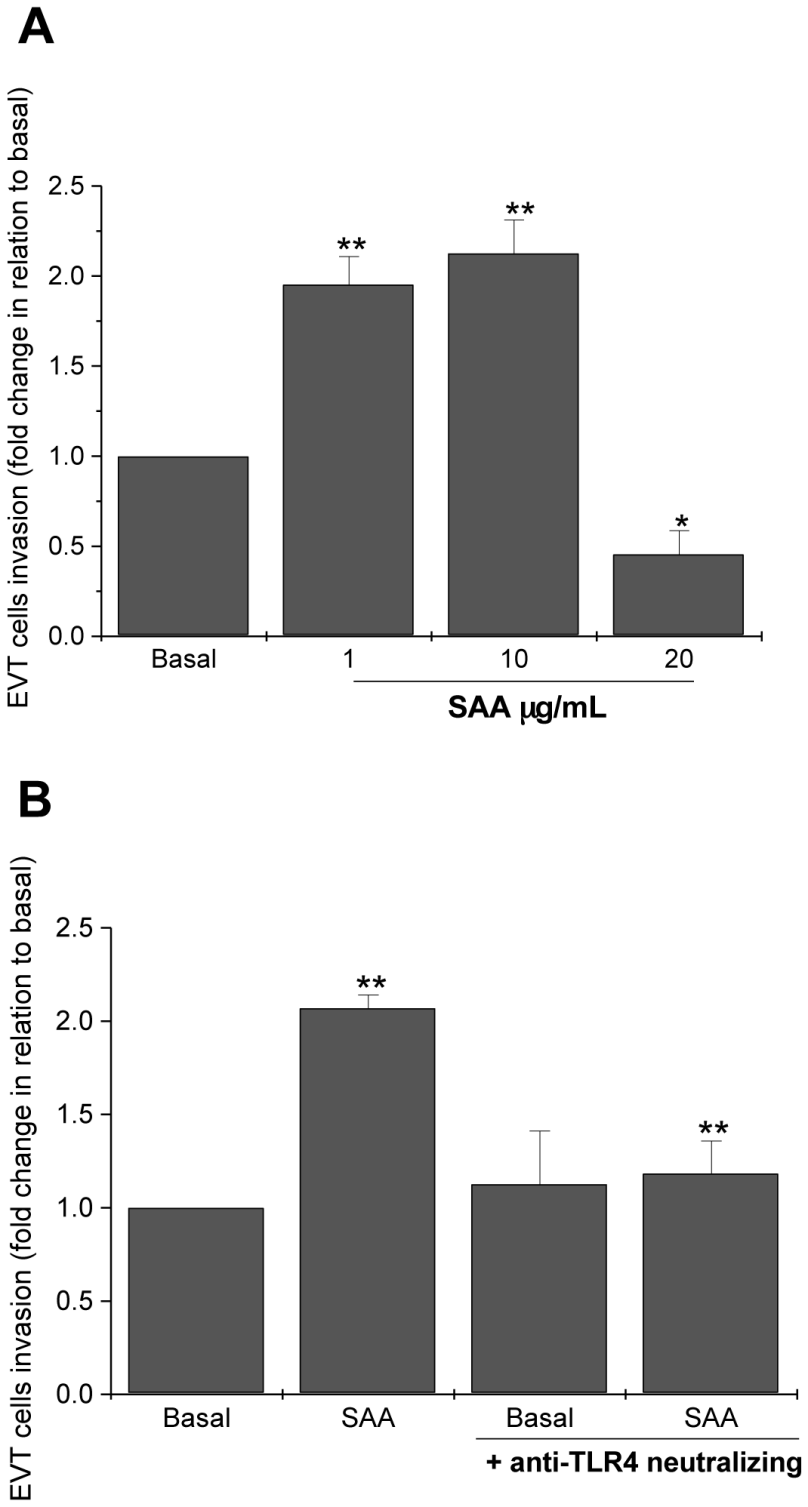
Effects of SAA on EVT cells. (A) SAA dependent on concentration affected the invasiveness of EVT cells in a Matrigel-coated transwell assay cultured for 48 h. (B) Inhibitory effect of a TLR4-neutralizing antibody on SAA-induced invasion of EVT cells. The data represent the median ± SE of four independent experiments performed in duplicate. *p<0.05 *vs*. basal; **p<0.01 *vs*. basal and treatment with the TLR4-neutralizing antibody.

To confirm that SAA-induced BeWo cell invasion also occurred in primary cells, we used term placentae as a source of EVT cells. EVT cells were isolated from the basal plate, stimulated with SAA at concentrations of 1, 10 and 20 µg/mL and incubated for 48 h on Matrigel-coated inserts. SAA treatment increased the invasiveness of term EVT cells at 1 and 10 µg/mL, but inhibited invasion at concentration of 20 µg/mL (**Fig. 3A**). Furthermore, we verified that SAA-induced EVT cell invasion was also dependent on TLR4, as the TLR4-neutralizing antibody showed an inhibitory effect (**Fig. 3B**).

### Expression of SAA in Trophoblastic Cells

**Figure 4:**
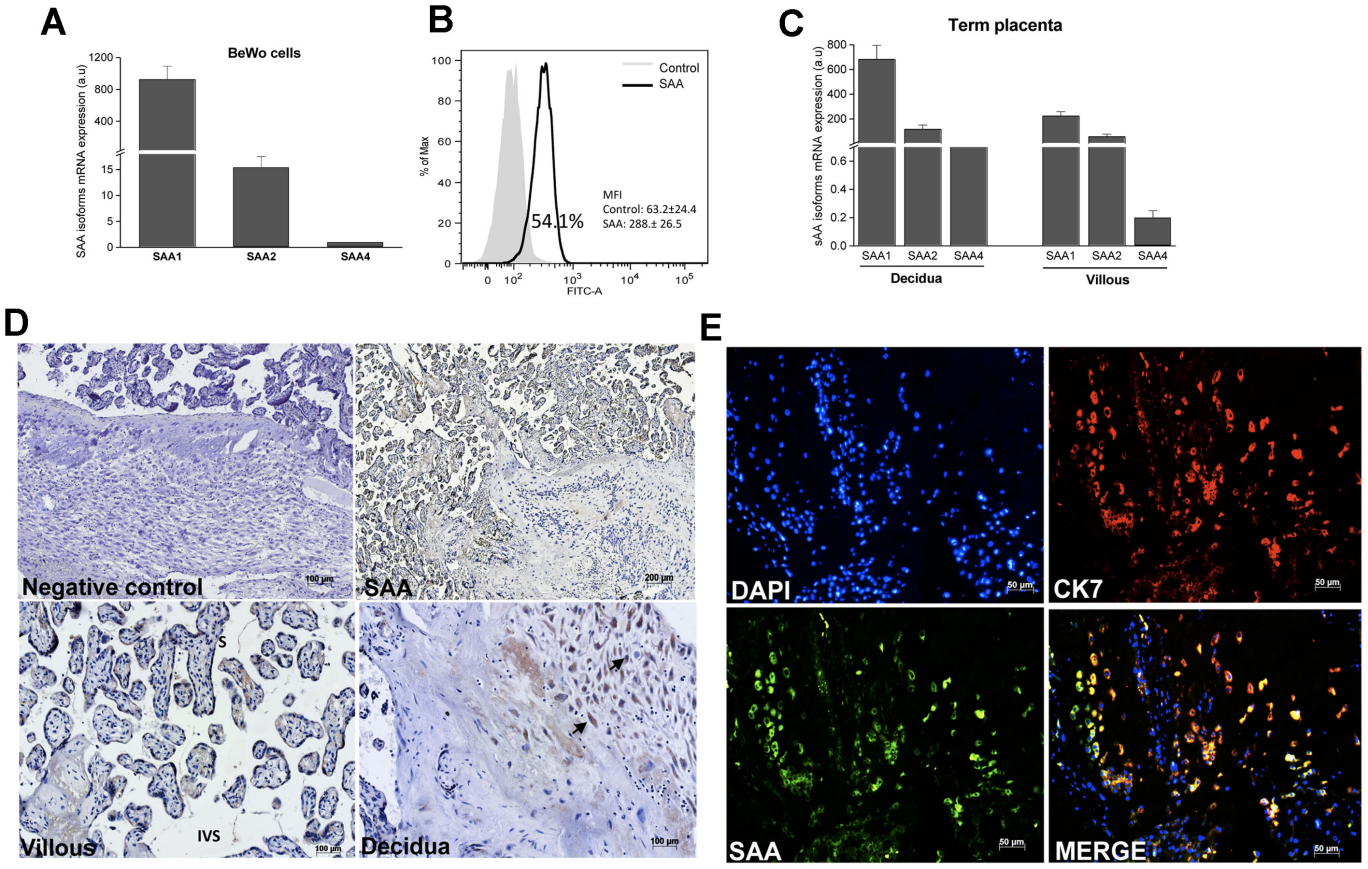
Expression of SAA. (A, C) mRNA expression of *SAA* isoforms in BeWo cells and term placenta, respectively. The data represent the median ± SE of four independent experiments. (B) SAA-positive BeWo cells. (D) The photomicrographs show the immunolocalization of SAA in the term placenta. Negative control (upper left); SAA staining (upper right); SAA was detected in syncytiotrophoblast (S) at villous side (lower left); and in decidual cells and trophoblasts at decidua side (arrowhead, lower right); IVS – intervillous space; (E) Immunofluorescence for SAA and CK7 at decidual side of term placenta. The data are representative of three independent experiments.

Given the ability of EVT cells to respond to SAA, we evaluated whether these cells expressed SAA protein constitutively. It was observed that BeWo cells constitutively expressed mRNA for the SAA1, SAA2 and SAA4 isoforms (**Fig. 4A**), and SAA protein was also detected. Flow-cytometric analysis of permeabilized BeWo cells showed that 54% of the cells expressed SAA, as there was a 5-fold increase in the fluorescence intensity when an anti-SAA antibody was used compared to the control antibody (**Fig. 4B**). Expression of SAA mRNA and protein was also detected in placental tissue. Both the decidual and villous sides of the placenta expressed the mRNAs for all SAA isoforms (**Fig. 4C**). Additionally, immunohistochemistry analysis showed that the syncytiotrophoblast was positive for SAA at the chorionic villi (**Fig. 4D**). In the decidual basal plate, apparently, SAA was detected in both decidual and EVT cells (**Fig. 4D**). To distinguish EVT cells among other cells types present in basal plate it was used immunofluorescence staining for cytokeratin-7 (CK7), a classical marker of trophoblast cells. Cells from the decidual basal plate were stained with anti-SAA or anti-CK7. By double staining assay we verified that CK7 positive cells were also labeled for SAA (**Fig. 4E**), indicating that trophoblast cells are producers of SAA.

## Discussion

The trophoblast is derived from the external trophectoderm layer of the blastocyst and differentiates through either the villous or extravillous trophoblast pathway [14]. In this study, we showed that SAA treatment promotes trophoblast invasion in a Matrigel-coated transwell assay, which is accompanied by an increase in both the mRNA expression and activity of metalloproteases. This invasive phenotype of BeWo cells is a characteristic of differentiation into extravillous trophoblast. Also, TLR4 was suggested to be involved in SAA-induced invasion. While SAA clearly affected the invasiveness of BeWo cells, it did not alter cell fusion observable by morphological change, one of the markers of syncytialization [23]. In fact, depending on its concentration, SAA promoted a decrease in the secretion of βhCG, a biochemical marker of syncytialization [23]. The possible impairment of syncytialization caused by SAA reminds that promoted by another inflammatory mediator, the TNF-α, in trophoblast cells [24].

Using fully differentiated EVT cells isolated from term placentae, we confirmed that SAA, up to 10 µg/mL, also increased trophoblast invasiveness ability in a TLR4-dependent manner. The inhibition of invasiveness at higher SAA concentration may indicate a possible reversed effect of SAA feasibly active *in vivo* during acute phase conditions in which the serum concentration of SAA may increase up to 1000-fold [1]. It is currently known that elevated levels of proinflammatory mediators, such as TNF-α, leads to a failed in trophoblast invasion [25], [26]. Both, unbalance in the production of proinflammatory mediators and failure in trophoblast invasion are found in preeclampsia and others placental disorders [27], [28]. In addition, it may be considered that hypoxia is associated with these placental disorders [28] and also could induce the production of SAA [29].

It is well known that successful pregnancy requires EVT invasion into the uterine decidua and the inner third of the myometrium, along with remodeling of the spiral arteries [14]. This process is closely associated with MMP-2 and MMP-9, as these are the major proteases involved in the invasive EVT phenotype [16]. Studies have shown that metalloproteases are modulated by SAA in a variety of different cell types. For instance, SAA is able to induce the production of MMP-2 and MMP-3 in synovial fibroblasts [30], MMP-9 activity in monocytic cells [31] and MMP-2 and 9 activity in glioma cell lines [13]. The increased activity of metalloproteases observed in trophoblast cells stimulated with SAA in the present study is consistent with both the expected action of SAA and the enhanced invasiveness of trophoblast cells on Matrigel. Moreover, the effect of SAA on trophoblast cell invasion was similar to that induced by other molecules that are expressed concurrently in the placental environment, such as adiponectin [32] and immunoregulatory molecules, such as IL-6 [33] and IL-8 [34]. It is also important to consider the possibility that other known functions of SAA that were not investigated here could be associated with trophoblast invasion, including modulation of integrins [35] and interactions with the ECM [36], which are considered to be the hallmarks of the invasive EVT phenotype [37], [38].

Considering that SAA is a ligand of a diversity of receptors, such as TLRs, RAGE and FPRs receptors [18], the effects of SAA on placental tissue may be the result of the binding of SAA to more than a single receptor type. Here, we showed that TLR4 plays a role in the increased invasiveness of trophoblastic cells treated with SAA, highlighting an additional contribution among the pleiotropic biological phenomena in which TLR4 is involved. In murine peritoneal macrophages, the association of SAA with TLR4 induces the production of inducible nitric oxide synthase (iNOS) and, hence, nitric oxide (NO) [10]. It was recently shown that TLR4 is involved in the invasiveness of some tumor cells triggered by endogenous and exogenous ligands [39], [40].

Although the liver is considered the main source of the acute-phase isoforms of SAA, several extra-hepatic tissues, such as breast, skin and adipose tissue, are also considered producers of SAA [41]. In fact, the majority of SAA synthesized during homeostatic conditions originates from adipose tissue [42]. Additional cell types have also been identified as important sources of SAA, including leukocytes [2], endothelial cells [43], synoviocytes [30] and glioma cells [13]. In these cells, the presence of SAA is related to the production of immunoregulatory mediators [8]–[10], chemotaxis [44], angiogenesis and migration [45], [46]. Furthermore, Kovacevic and colleagues showed that the levels of SAA1 and SAA2 transcripts are increased in first trimester trophoblast during pregnancy at 10 and 12 weeks, suggesting that SAA plays a role during early fetal development [6].

Here, we demonstrated that SAA mRNA and protein are expressed in the cells that comprise the term placenta, including syncytiotrophoblast, cytotrophoblast and decidual cells, as well as in the BeWo cell line. SAA presented the same mRNA expression profile in BeWo cells and in both the villous and decidual sides of the term placenta, with predominance of the isoform encoding the acute-phase protein SAA1 and SAA2 being observed. Similarly it has been described in adipose tissue [12] and tumor cells [13]. Additionally, SAA protein was detected in the intracellular compartment of trophoblast cells. In the villous side of the term placenta, SAA was localized in syncytiotrophoblast, which form a layer that secretes placental hormones and exchanges gases and nutrients between the maternal and fetal circulation [47]. In the decidual basal plate, SAA was observed in both extravillous trophoblast and decidual cells, which are responsible for maternal-fetal tolerance [48].

The presence of SAA in first trimester trophoblast [6], together with our data showing that third trimester placental cells are able to express and respond to SAA, suggests that this protein may exhibit an important function during the progression of pregnancy. The role of SAA may be related to placentation through the modulation of trophoblast invasion, which is necessary in the early stages of pregnancy, as well as to maintaining the balance between pro- and anti-inflammatory cytokines, which is a well-known function of SAA required for the maintenance of maternal-fetal tolerance. Furthermore, it should be noted that the presence of SAA in third trimester placental cells may be related to the modulation of IL-1β, IL-6 and IL-8 [49] and metalloproteases [50], which trigger the onset of labor and delivery.

In summary, this study described a critical function for SAA in the placental environment: the modulation of trophoblast cell invasiveness. Additionally, we present the first evidence that activation of TLR4 by SAA may be involved in other processes beyond the production of NO and other immunoregulatory factors.
